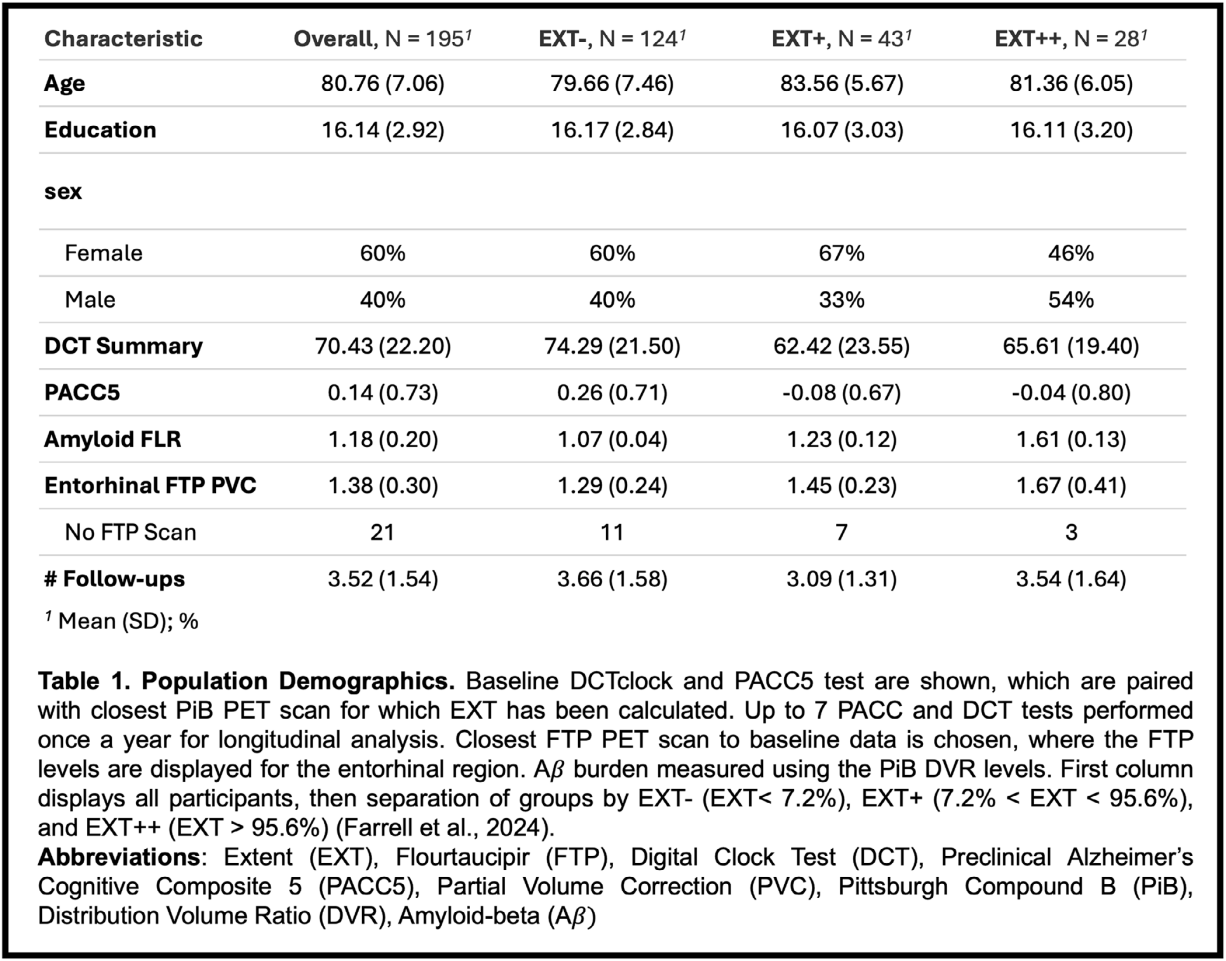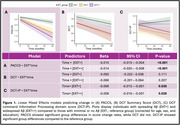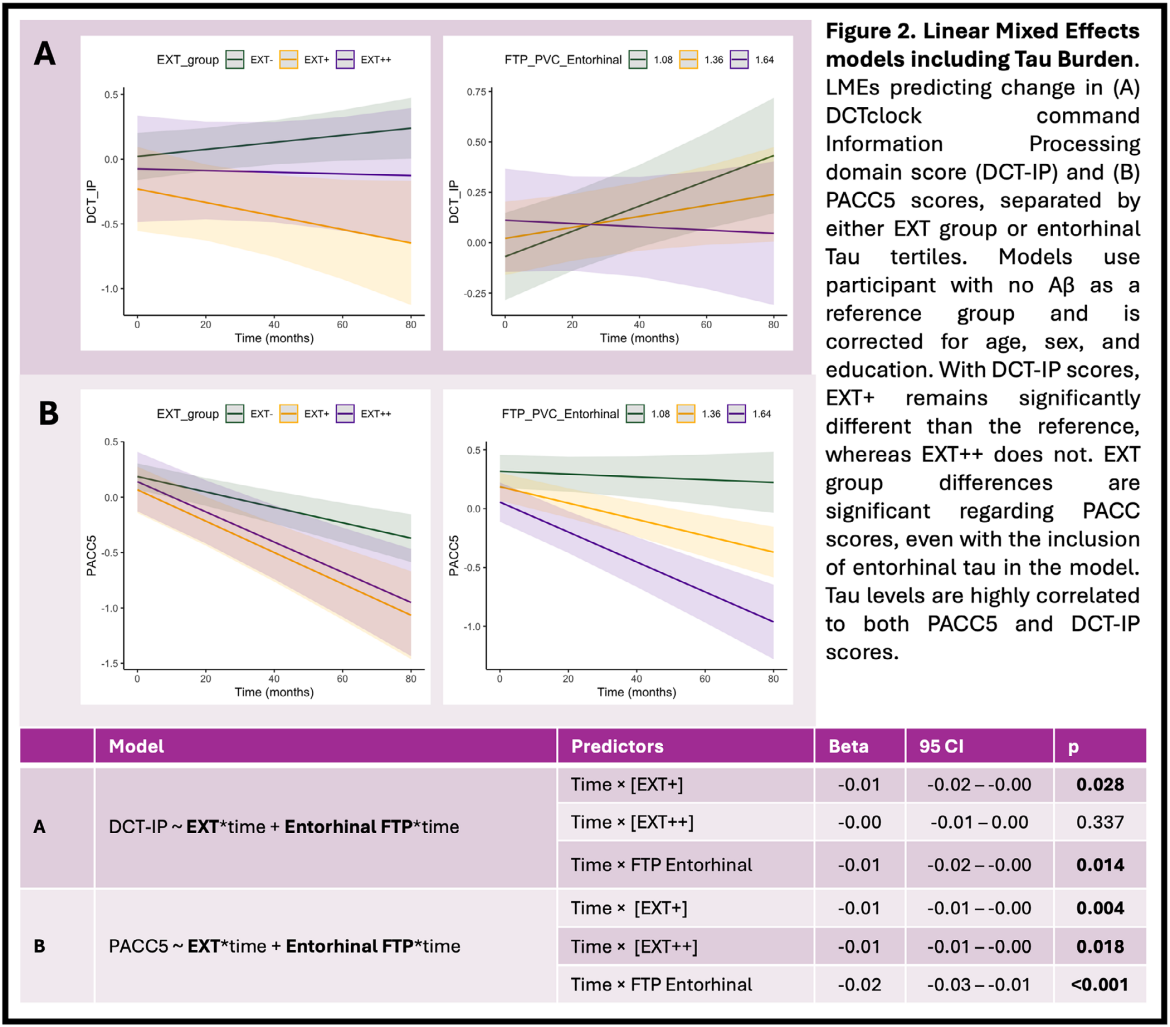# Baseline amyloid spatial extent in combination with tau burden captures cognitive decline, measured digital clock drawing test and PACC5, in preclinical Alzheimer's disease

**DOI:** 10.1002/alz70857_106687

**Published:** 2025-12-26

**Authors:** Jackson C Thompson, Jessie Fanglu Fu, Michelle E. Farrell, Emma G Thibault, Elliott Slade, Grace A Del Carmen Montenegro, Marina Rodriguez Alonso, Talia L. Robinson, Roos J Jutten, Dana Penney, Randall Davis, Reisa A. Sperling, Julie C Price, Dorene M. Rentz, Keith A. Johnson

**Affiliations:** ^1^ Massachusetts General Hospital, Harvard Medical School, Boston, MA, USA; ^2^ Athinoula A Martinos Center for Biomedical Imaging, Massachusetts General Hospital, Harvard Medical School, Charlestown, MA, USA; ^3^ Massachusetts General Hospital, Boston, MA, USA; ^4^ Lahey Hospital and Medical Center, Lexington, MA, USA; ^5^ Digital Cognition Technologies, Waltham, MA, USA; ^6^ MIT Computer Science And Artificial Intelligence Laboratory, Cambridge, MA, USA; ^7^ Harvard Medical School, Boston, MA, USA

## Abstract

**Background:**

Digital cognitive assessments have potential to improve detection of early cognitive changes in emerging Alzheimer's disease (AD) pathology in preclinical AD. Spatial extent (EXT) measures the spread of amyloid‐b (Ab) and is potentially more sensitive to early Ab burden than the traditional measure of neocortical Ab level. Previously, we observed that worsening digital clock drawing test (DCTclock) performance was cross‐sectionally associated with spreading amyloid and greater entorhinal tau in cognitively normal (CN) participants. We sought to evaluate these relationships longitudinally.

**Method:**

195 CN older adults from the Harvard Aging Brain Study were included with annual DCTclock and PACC5 assessments for up to 6 years (Table 1). DCTclock measures included a composite summary score (DCT) and 4 subscores (information processing, spatial reasoning, simple motor, drawing efficiency) for both command and copy versions. Baseline amyloid ([^11^C]Pittsburgh Compound B) and entorhinal tau (Flortaucipir SUVR, *n* = 174) PET were measured. Abspread was estimated as the neocortical spatial extent (EXT, %PiB+ using region‐specific thresholds) and grouped into 3 EXT stages: EXT‐ (EXT<7.2%, no Ab), EXT+ (7.2%EXT<95.6%, spreading Ab), EXT++ (EXT95.6%, widespread Ab). A series of linear mixed effect models assessed the effects of EXT group and entorhinal tau on PACC5 and DCTclock over time, corrected for age, sex, and education.

**Result:**

Individuals in the EXT+ and EXT++ groups showed a significantly faster cognitive decline on PACC5 and DCTclock command information processing subscore (DCT‐IP) compared to EXT‐ (Figure 1). When including ERC tau, elevated ERC tau burden and EXT+/EXT++ independently contributed to a faster decline in PACC5, whereas faster decline in DCT‐IP was significant in the EXT+ group only (Figure 2).

**Conclusion:**

Longitudinal assessment with DCTclock captured unique information about cognitive decline in the early stages of the spread of amyloid. However, the PACC remained a superior measure of the more severe cognitive decline associated with amyloid and tau pathology in later stages of preclinical AD.